# Assessment of Public Awareness and Practices Regarding Tinea Pedis Among the Saudi Arabian Population

**DOI:** 10.7759/cureus.59753

**Published:** 2024-05-06

**Authors:** Hanan A AlKaabi, Nouf Alhammadi, Marwah AL-Jallal, Ward M Malibari, Rahaf S Al Jallal, Abdulmalik S Almarshad, Fahad H Binshalhoub, Amirah Albalawi, Tahani A Adam, Alaa H Al-Khairat, Mohammed H Albarqi, Ahmad Assiri, Saleh S Al Qahtani, Anas A Sayegh, Alhassan H Hobani

**Affiliations:** 1 College of Medicine, King Khalid University, Abha, SAU; 2 Rheumatology, King Khalid University, Abha, SAU; 3 College of Medicine, Umm Al-Qura University, Makkah, SAU; 4 Radiology, King Khalid University, Abha, SAU; 5 College of Medicine, Qassim University, Buraydah, SAU; 6 Medicine and Surgery, Imam Muhammad Ibn Saud Islamic University, Riyadh, SAU; 7 College of Medicine, University of Tabuk, Tabuk, SAU; 8 Statistics and Operation Research, Qassim University, Buraydah, SAU; 9 College of Medicine, Jazan University, Jazan, SAU; 10 Dentistry, King Khalid University, Abha, SAU; 11 Dermatology, Jazan University, Jazan, SAU; 12 Emergency Medicine, King Fahd Hospital of the University, Najran, SAU; 13 Surgery, Jazan University, Jazan, SAU

**Keywords:** foot hygiene, feet examination, saudi arabia, perception, awareness, tinea pedis

## Abstract

Background: Tinea pedis, commonly known as athlete's foot, is a fungal infection affecting the skin of the feet, primarily between the toes. Despite being a common condition, there may be gaps in knowledge and practices regarding its transmission, risk factors, and treatment options among the general population.

Methods: This study adopted a cross-sectional research design. The study involved 2371 adult population in Saudi Arabia. The questionnaire was distributed online through social media means. Data was analyzed using IBM SPSS Statistics for Windows, Version 27 (Released 2020; IBM Corp., Armonk, New York, United States).

Results: A significant portion (66.1%) of respondents reported not examining their feet regularly. The majority (80.7%) of the respondents reported regular use of nail scissors as the common practice. The results further revealed that the majority of respondents (71.7%) were not aware of tinea pedis. Similarly, a large proportion (77.3%) of respondents were unaware of the risk factors associated with tinea pedis. However, among those aware, the most recognized risk factor was diabetes mellitus (82.3%), followed by peripheral arterial disease (37.1%), and immunocompromised conditions like HIV (31.3%). There was a significant association (p = 0.001) between regular foot examination practices and awareness of tinea pedis.

Conclusion: In conclusion, there is low awareness regarding tinea pedis among the Saudi Arabian population. Only a small proportion demonstrated good knowledge of the condition and its symptoms. However, there is a relatively higher awareness of specific risk factors such as diabetes mellitus and peripheral arterial disease. Hence, it is imperative to enhance education and awareness campaigns to address the gaps in understanding tinea pedis, its associated risk factors, and symptoms, particularly among individuals engaged in physical activities and those who regularly wear sports shoes.

## Introduction

Tinea pedis, commonly known as athlete's foot, develops when dermatophytes infiltrate the skin of the feet. It is mainly spread through direct contact with the organism, often while walking barefoot. Symptoms typically manifest in the interdigital spaces of the toes but can also affect the soles and edges of the feet [1]. Tinea pedis manifesting symptoms include itching, scaling, and burning skin, often leading to cracking and blistering. Tinea pedis thrive in moist environments, particularly in pools and public showers [2,3]. Prevention measures include maintaining dry and clean feet, consistently wearing sandals, and avoiding enclosed shoes and socks [4]. Seeking prompt medical attention for treatment upon suspicion of the condition is crucial to prevent its spread. Untreated tinea pedis can result in significant complications, notably impacting a person's appearance. Individuals with diabetes mellitus have an elevated risk of getting tinea pedis [5,6]. Topical antifungals represent the safest treatment for tinea pedis. However, recurrence is frequent, necessitating prolonged treatment in many cases [7].

A study by Song and Li demonstrated that 15.8% of tinea pedis patients did not know about the condition before their infection. Another survey by Doğan et al. highlights that public awareness regarding tinea pedis remains low within the population despite its prevalence [8]. The tinea pedis is believed to be prevalent in Saudi Arabia but the existing knowledge gap necessitates the urgent need for a public awareness campaign aimed at improving the awareness level in the country [8,9]. The studies underscore the pressing necessity for educating the population about the risk factors and preventive interventions to mitigate the spread of the infection. Given the intersection of these conditions, proactive measures are imperative to effectively address the impact of tinea pedis on the population, particularly in light of its high prevalence level [9].

It is essential to develop a comprehensive public awareness education campaign regarding tinea pedis symptoms, preventive measures, and risk factors [10,11]. Tinea pedis is a common fungal infection affecting the feet, with the potential to cause discomfort, pain, and complications if left untreated. Despite being widespread, there is an insufficiently comprehensive understanding of public awareness and practices concerning tinea pedis among the Saudi population. Investigating these aspects is crucial for gaining insights into the level of knowledge, preventive measures, and treatment practices adopted by individuals in Saudi Arabia.

## Materials and methods

Study design

This research utilized a cross-sectional design and was carried out between October 2023 and March 2024. It encompassed Saudi Arabia's adult population. Its primary aim was to evaluate the public's awareness and perception concerning tinea pedis within the context of Saudi Arabia.

Inclusion and exclusion criteria

The inclusion criteria encompassed adults of both genders residing within the confines of Saudi Arabia. Excluded from the study were individuals under the age of 18 and those living outside the borders of Saudi Arabia.

Sampling technique

The sampling technique used in the study was convenient sampling. Respondents were enrolled in the study based on their availability and willingness to participate during the time of data collection. At the outset of the study, respondents were invited to declare their desire to take part.

Sample size

To determine the sample size, we used the Cochrane sample size approach, which is given below.

n = Z2 (1 − p)/d2. Where n is the sample size, Z is the critical statistic for a 95% confidence interval the anticipated prevalence was set at 15%, and d is the margin of error, set at 0.02. The minimum acceptable sample size was 1832, but we used a larger sample of 2371 respondents.

Data collection tools and procedures

The researchers developed a questionnaire that aligned it with the study's aims and objectives and drew from relevant literature. The piloting of the questionnaire involved 10 respondents to assess its feasibility, understandability, and readability. The respondents involved in the pilot study were not included in the main study. Following adjustments based on pilot feedback, the questionnaire was distributed online to respondents via WhatsApp and Telegram groups. It covered demographics, foot hygiene practices, and awareness sections.

Data analysis

Following the completion of the data collection phase, the data underwent a thorough data cleaning exercise. The data cleaning process encompassed various tasks such as identifying and removing duplicate entries, scrutinizing for outliers, and addressing any missing data points. Later, the data was coded and entered into IBM SPSS Statistics for Windows, Version 27 (Released 2020; IBM Corp., Armonk, New York, United States). Categorical variables were presented in terms of count and frequencies, and the chi-square test was used to determine the association between categorical variables with a predetermined significance level set at p < 0.05. This comprehensive analytical approach aimed to extract meaningful insights and discern patterns within the dataset.

Ethical considerations

For this study, ethical approval was obtained from the Research Ethics Committee at King Khalid University (IRB No: 2023-1107). A number of ethical considerations were taken into consideration, such as maintaining confidentiality and ensuring informed consent. The participants were fully informed about the study's purpose, procedures, risks, and benefits before agreeing to participate. Participants had to provide voluntary and informed consent without coercion. The researchers assured the respondents to safeguard the privacy of participants by protecting their identity and sensitive information.

## Results

Table 1 provides demographic information about the study respondents. Among the participants, the majority were female; 1443 (60.99%) were female. Age distribution ranged from below 20 years (11.4%) to above 60 years (5.4%). Most of the participants were Saudi nationals (2168 (91.4%)) with 203 (8.6%) being non-Saudi. Residency was spread across different regions, with the Western region having the highest representation at 740 (31.2%). Regarding education, the majority held university degrees 1557 (65.7%), followed by secondary education 534 (22.5%). Occupation varied, with students comprising the largest group (1031 (43.5%)) followed by others (695 (29.3%)). Body mass index (BMI) ranged from less than 18.5 (9.2%) to more than 30 (22.4%). Smoking prevalence was 415 (17.5%), while 1956 (82.5%) were non-smokers.

**Table 1:** Socio-demographic variables of the respondents Socio-demographic information presented in frequencies (n) and percentages (%).

Variables	Category	Frequency (percentage)
Gender	Male	928 (39.9%)
	Female	1443 (60.99%)
Age	Below 20 years	270 (11.4%)
	20-30 years	1109 (46.8)
	31-40 years	456 (19.2%)
	41-50 years	249 (10.5%)
	51-60 years	159 (6.7%)
	Above 60 years	128 (5.4%)
Nationality	Saudi	2168 (91.4%)
	Non-Saudi	203 (8.6%)
Residency	Eastern Region	412 (17.4%)
	Middle Region	631 (26.6%)
	Northern Region	94 (4.0%)
	Southern Region	494 (20.8%)
	Western Region	740 (31.2%)
Education	Primary	139 (5.9%)
	Secondary	534 (22.5%)
	University	1557 (65.7%)
	Postgraduate	141 (5.9%)
Occupation	Students	1031 (43.5%)
	Teacher	262 (11.1%)
	Military	31 (1.3%)
	Housewife	61 (2.6%)
	Retired	41 (1.7%)
	Engineer	145 (6.1%)
	Doctor	105 (4.4%)
	Others	695 (29.3%)
BMI	Less than 18.5	219 (9.2%)
	18.5-24.9	1070 (45.1%)
	25-29.9	552 (23.3%)
	More than 30	530 (22.4%)
Smoking	Yes	415 (17.5%)
	No	1956 (82.5%)

Table 2 shows the level of foot care habits of the respondents. A total of 803 (33.9%) respondents reported examining their feet regularly, while the majority (1568 (66.1%)) did not. The majority of 2179 (81.5%) indicated a regular use of nail scissors. Regarding socks usage, a slight majority of 1515 (56.7%) of the respondents reported wearing socks regularly, whereas a majority of 1869 (69.9%) of them wear socks during winter. The majority of respondents (1745 (73.6%)) reported washing their feet three times or more daily, while 626 (26.4%) reported washing them less than three times daily. 

**Table 2:** Foot hygiene practices among the respondents Foot hygiene practices presented in frequencies (n) and percentages (%).

Variables	Category	Frequency
Do you examine your feet regularly?	Yes	803 (33.9%)
	No	1568 (66.1%)
Do you use nail scissors regularly?	Yes	1913 (80.7%)
	No	458 (19.3%)
Do you wear socks regularly?	Yes	1351 (57.0%)
	No	1020 (43.0%)
Wearing socks	Rarely	723 (30.5%)
	During winter	1648 (69.5%)
	Frequently	723 (30.5%)
How many times do you wash your feet daily?	Three times or more	1745 (73.6%)
	Less than three times	626 (26.4%)

Table 3 illustrates the respondents' awareness level of tinea pedis. The majority (1909 (71.4%)) of the respondents were not familiar with this condition. Regarding the awareness of the condition's risk factors, the majority of 2063 (77.2%) were not aware of tinea pedis' risk factors. About 1383 (51.8%) of the respondents demonstrated that Diabetes Mellitus (DM) is the risk factor, 255 (9.5%) reported DM, and immunocompromised (HIV) is the risk factor of the condition. Most of the study participants (2019 (75.6%)), were not aware of tinea pedis symptoms. About 842 (31.5%) know that only pain is a symptom of tinea pedis. Most of the respondents (962 (36.0%)) sometimes work out. The majority (893 (33.4%)) often wear sports shoes for four to eight hours. Last, the majority of the participants (2211 (82.7%)) do not smoke.

**Table 3:** Awareness about tinea pedis Awareness items are presented in frequencies (n) and percentages (%). h: hours; DM: diabetes mellitus

Variables	Category	Frequency (percent)
Are you aware of athlete’s foot?	Yes	671 (28.3%)
	No	1700 (71.7%)
Are you aware of athlete’s foot risk factors?	Yes	538 (27.7%)
	No	1833 (77.3%)
Risk factors of athlete’s foot include:	DM	1951 (82.3%)
	Immunocompromised HIV)	741 (31.3%)
	Peripheral arterial disease	879 (37.1%)
Are you aware of athlete’s foot symptoms?	Yes	578 (24.4%)
	No	1793 (75.6%)
Symptoms of athlete’s foot includes	Pain	1585 (66.8%)
	Itching	1164 (49.1%)
	Redness	1225 (51.7%)
Do you do workout	Never	296 (12.5%)
	Little	697 (29.4%)
	Sometimes	838 (35.3%)
	Usually	343 (14.5%)
	Always	197 (8.3%)
How often do you wear sport shoes?	Less than 2 h	645 (27.2%)
	2-4 h	610 (25.7%)
	4-8 h	801 (33.8%)
	More than 8 h	315 (13.3%)

Table 4 depicts the association between the practices of tinea pedis and its awareness. Notably, there is a statistically significant association (p < 0.001) between regular foot examination and the awareness of tinea pedis, with 333 (41.5%) respondents examining their feet regularly. Similarly, there are significant associations between the regular use of nail scissors and foot examination (p < 0.001), as well as between the regular use of socks and foot examination (p < 0.001). There is a significant association (p < 0.001) between the frequency of wearing socks and the awareness of tinea pedis, with a higher proportion of respondents who wear socks regularly being aware of the condition compared to those who do not. Moreover, there is a significant association (p < 0.001) between the frequency of washing their feet daily and awareness of tinea pedis, with a higher proportion of respondents who wash their feet three times or more daily being aware of the condition. However, the association between the frequency of washing feet daily and awareness of tinea pedis is not statistically significant (p = 0.293).

**Table 4:** Association between the practices towards foot hygiene and awareness about tinea pedis Data has been presented in frequencies (n) and percentages (%). The chi-square test was used to determine significance. The p-value was considered statistically significant at p-value < 0.05.

Variable	Category	Are you aware of tinea pedis?		p-value
		Yes	No	
Do you examine your feet regularly?	Yes	333 (41.5%)	470 (58.5%)	
	No	338 (21.6%)	1230 (78.4%)	0.001
Do you use nail scissors regularly?	Yes	581 (30.4%)	1332 (69.4%)	
	No	90 (19.7%)	368 (80.3%)	0.001
Do you wear socks regularly?	Yes	445 (32.9%)	906 (67.1%)	
	No	226 (22.2%)	794 (77.8%)	0.001
Wearing socks	Rarely	156 (21.6%)	567 (88.4%)	
	During Winter	515 (31.3%)	1133 (68.7%)	0.001
How many times do you wash your feet daily?	Three times or more	504 (28.9%)	1241 (71.1%)	
	Less than three times	167 (26.7%)	459 (73.3%)	0.293

Table 5 provides the relationship between the respondent’s socio-demographic information and level of knowledge about tinea pedis' condition. The study established a statistically significant relationship between gender, residency, education, and occupation with p values < 0.05 (0.004, 0.001, 0.002, and 0.001, respectively) and knowledge about the tinea pedis condition. On the other hand, there was no statistically significant association between age, nationality, BMI, and the level of knowledge of the condition with a p-value > 0.05.

**Table 5:** The association between socio-demographic information and level of knowledge about tinea pedis Data has been presented in frequencies (n) and percentages (%). The chi-square test was used to determine significance. The p-value was considered statistically significant at p-value < 0.05.

Variable	Category	Knowledge levels		p-value
		Poor	Good	
Gender	Male	773 (74.6%)	263 (25.4%)	0.004
	Female	1909 (69.4%)	763 (30.6%)	
Age	Below 20 years	208 (71.2%)	84 (28.8%)	0.085
	20-30 years	865 (70.2%)	367 (29.8%)	
	31-40 years	391 (76.2%)	122 (23.8%)	
	41-50 years	223 (72.6%)	844 (27.4%)	
	51-60 years	126 (66.3%)	64 (33.7%)	
	Above 60 years	96 (69.6%)	42 (30.4%)	
Nationality	Saudi	1750 (71.3%)	706 (28.7%)	0.462
	Non-Saudi	159 (73.6%)	57 (26.4%)	
Residency	Eastern Region	380 (72.4%)	145 (27.6%)	
	Middle Region	515 (78.0%)	145 (22.0%)	
	Northern Region	79 (66.9%)	39 (33.1%)	
	Southern Region	357 (64.3%)	198 (35.7%)	0.001
	Western Region	578 (64.3%)	236 (29.0%)	
Education	Primary	106 (66.7%)	53 (33.3%)	0.002
	Secondary	422 (69.4%)	186 (30.6%)	
	University	1276 (72.8%)	477 (27.2%)	
	Postgraduate	105 (69.1%)	47 (30.9%)	
Occupation	Students	783 (68.6%)	359 (31.4%)	0.001
	Teacher	221 (71.3%)	89 (28.7%)	
	Military	34 (85.0%)	6 (15.0%)	
	Housewife	73 (71.6%)	29 (28.4%)	
	Retired	39 (76.5%)	12 (23.5%)	
	Engineer	127 (77.9%)	36 (22.1%)	
	Doctor	52 (46.4%)	60 (53.6%)	
	Others	580 (77.1%)	172 (22.9%)	
BMI	Less than 18.5	166 (70.0%)	71 (30.0%)	0.31
	18.5-24.9	873 (72.6%)	330 (27.4%)	
	25-29.9	456 (72.5%)	173 (27.5%)	
	More than 30	414 (68.7%)	189 (31.3%)	
Smoking	Yes	415	17.50%	0.45
	No	1956	82.50%	

## Discussion

The aim of this study was to determine the public awareness level, perception, and risk factors of tinea pedis condition in Saudi Arabia. The study findings reveal diverse foot hygiene practices and lifestyle habits among the study participants. The majority of the respondents (1769 (66.2%)) do not examine their feet regularly. The habit of not examining feet among the respondents aligns with the trend observed in numerous studies that reflect the lack of consistent examination of feet [12]. Furthermore, the majority (2179 (81.5%)) reported regular use of nail scissors, suggesting that the use of scissors is the best hygiene and grooming habit among the study participants. The study further established that most respondents (1351 (57.0%)) wear socks regularly, and 1648 (67.5%) wear socks during winter. The high frequency of using nail scissors suggests that grooming practices may be more widely adopted compared to other aspects of foot care, such as regular foot examination. A study by Alhammadi et al. revealed that maintaining foot hygiene by using nail scissors and wearing socks regularly is a crucial element in preventing tinea pedis [13].

The results indicate limited awareness, with only 24.4% of respondents indicating good awareness. The most recognized symptoms among those who were aware included pain (66.8%), itching (49.1%), and redness (51.7%). As far as lifestyle is concerned, there are varying levels of physical activity, with a significant portion engaging in workouts either sometimes (35.3%) or little (29.4%). When it comes to footwear habits, the majority wear sports shoes for extended periods, with 33.8% wearing them for 4-8 hours and 27.2% wearing them for less than 2 hours. These findings highlight the need for increased education and awareness campaigns regarding tinea pedis, its risk factors, and its symptoms, particularly among individuals who engage in physical activities and wear sports shoes regularly. A study by Khalifa et al. revealed that a significant (78%) proportion of tinea pedis patients were not aware of the conditions’ symptoms before they were infected [14].

Certain foot hygiene practices like the use of nail scissors, regularity of wearing socks, and wearing socks during winter months revealed a statistically significant relationship with awareness of tinea pedis (p < 0.05). Most respondents who reported engaging in these practices also indicated awareness of tinea pedis compared to those who did not. Specifically, among respondents who examine their feet regularly, 41.5% were aware of tinea pedis, whereas among those who do not, only 21.6% were aware. Similarly, among individuals who reported regular use of nail scissors, 30.4% were aware of tinea pedis, compared to 19.7% among those who did not use nail scissors regularly. The pattern continues with wearing socks regularly and wearing socks during winter months, where higher awareness percentages were observed among those who reported these habits. These findings suggest that certain foot care habits, such as foot examination, regular use of nail scissors, and consistent sock-wearing habits, may be associated with higher awareness of tinea pedis. A similar study conducted in the Hail region in the Kingdom of Saudi Arabia (KSA) found low awareness (34.82%) but a positive correlation between practices and awareness [15].

The study findings indicated that some social demographic variables, namely gender, education, and residency, exhibited a statistically significant relationship with tinea pedis awareness level among the participants (p < 0.05). Similarly, certain occupations, such as doctors, displayed higher knowledge levels compared to others, highlighting the potential influence of professional training and experience on health-related awareness. On the other hand, nationality and residency did not show statistically significant associations with knowledge levels, suggesting that knowledge of health-related issues may be relatively consistent across different demographic groups in these categories. A similar study done within Saudi Arabia revealed that gender, occupation, smoking, and residency were statistically significant with the tinea pedis awareness level among participants, with males, healthcare workers, and urban dwellers showing more knowledge about the condition [16]. Another study conducted in Tunisia revealed that age and the area of residence were important predictors of tinea pedis [17].

One significant limitation is the utilization of a cross-sectional study design. Although cross-sectional studies are adept at discerning associations between variables at a particular point in time, they inherently lack the ability to establish causation or monitor developments over time. Consequently, longitudinal or mixed-methods methodologies would yield more profound insights into the public awareness and practices of tinea pedis.

## Conclusions

The study reveals a concerning lack of awareness among the Saudi Arabian population, with a small proportion of respondents demonstrating knowledge of the condition and its symptoms. However, there is comparatively higher awareness of specific risk factors, notably diabetes mellitus and peripheral arterial disease. This study highlights the need for increased education and awareness campaigns to improve knowledge of tinea pedis, its risk factors, and symptoms, particularly among individuals engaged in physical activities and those who regularly wear sports shoes. Demographic variables like gender, education, residency, and occupation are significant predictors of awareness levels, suggesting the importance of targeted interventions tailored to specific demographic groups to enhance prevention and management strategies for tinea pedis in Saudi Arabia.
